# The Role of Body Image in Internalizing Mental Health Problems in Spanish Adolescents: An Analysis According to Sex, Age, and Socioeconomic Status

**DOI:** 10.3389/fpsyg.2019.01952

**Published:** 2019-08-22

**Authors:** Pilar Ramos, Concepción Moreno-Maldonado, Carmen Moreno, Francisco Rivera

**Affiliations:** ^1^Department of Developmental and Educational Psychology, Faculty of Psychology, University of Seville, Seville, Spain; ^2^Department of Experimental Psychology, Faculty of Psychology, University of Seville, Seville, Spain

**Keywords:** body image, body mass index, dieting, physical activity, internalizing symptoms, sex, age, socioeconomic status

## Abstract

During adolescence there is a relatively high prevalence of weight problems and eating disorders. Furthermore, body image plays an important role in weight control and eating behaviors as well as in mental health. This study analyses the influence of body mass index, perception of being overweight, and body image satisfaction (BIS) on internalizing symptoms related to mental health in adolescents. In addition, sex, age, socioeconomic status (SES), dieting, and physical activity are taken into consideration. This research is based on the international study *Health Behaviour in School-aged Children* (HBSC). The sample consists of 4531 Spanish adolescents from 13 to 18 years old. Participants were selected through random multi-stage sampling stratified by conglomerates. Two instruments were employed: the HBSC questionnaire and the Youth Self-Report (Achenbach System of Empirically Based Assessment, ASEBA). Results demonstrated that BIS –the emotional component related to body image– was the main predictor of adolescent internalizing symptoms. In addition, results show double-inequalities according to the interaction effects of sex, age, and SES. Likewise, interesting results are shown regarding how dieting behaviors to lose or gain weight/volume and physical activity relate to body image perception and satisfaction, as well as with internalizing symptoms. This study highlights important body image aspects relevant to intervention and prevention of internalized mental health problems in adolescence.

## Introduction

Adolescence is a period characterized by physiological, emotional, cognitive, and social changes that lead to a greater concern for physical appearance. The physical changes that accompany the onset of puberty demand a constant restructuring of the adolescent’s perception of their body, provoking an increased preoccupation for this image that in many cases leads to a decrease in self-esteem (Harter, 2006; Mills et al., 2011). The propensity of adolescents to be unhappy with their body image has been addressed in many studies, arguing that this problem with body image can threaten their health and wellbeing (Shagar et al., 2017).

Body image refers to the multifaceted psychological experience of embodiment that encompasses one’s body-related self-perceptions and self-attitudes, including thoughts, beliefs, feelings, and behaviors. Body image has often been defined as the self-perception of the physical self and the feelings and thoughts that result from that perception (Cash, 2004; Grogan, 2006). Disturbance in any of these domains is referred to as body image concerns or negative body image.

Previous findings have shown a relationship between body image concerns and the development of psychopathology in adolescence, especially with internalizing symptoms (IS) – i.e., anxiety, depression, or social withdrawal (Thompson et al., 1995; McClintock, 2001; Siegel, 2002; Ter Bogt et al., 2006; Patalay and Hardman, 2019)–, which in turn is a risk factor for the development of eating disorders (Graber and Sontag, 2009; Puccio et al., 2016). For example, a previous study of more than 7000 Dutch adolescents showed an association between deviance from normal weight and IS, however not with externalizing problems (Ter Bogt et al., 2006). According to the authors, “lower body mass index [BMI] scores relate to greater problems, more specifically to anxiety/depression and social problems, higher BMI is associated with withdrawnness. However, body weight perception is a more substantial predictor of adolescent distress (…). Perception of being too heavy is the most substantial predictor of a wide range of problems and both the overweight and the young people with normal BMI are affected” (p. 32).

Appraisals of physical development or body image are areas of self-evaluation that are particularly salient to adolescents and their risk for internalizing problems (Graber and Sontag, 2009). Children with higher BMI may feel discriminated against due to their image, which would lead them to present internalizing symptoms in the long-term. This is related to an increased awareness and internalization of social attitudes around weight throughout childhood, which is manifested in more psychological problems in adolescents with worse body image (Puhl and Latner, 2007). Specifically, body image concerns can often be attributed to social and cultural pressures (Bessenoff and Snow, 2006). According to the systematic review conducted by Sobrino-Bazaga and Rabito-Alcoón (2018), sociocultural factors had the most influence on body image dissatisfaction. In addition, having a body image close to the socially accepted ideal of beauty is conceptually associated with possessing other positive characteristics such as being a successful or healthy person (Tiggemann et al., 2000; Neziroglu et al., 2008; Mischner et al., 2013).

In explaining human development, and in this case adolescent development, certain theoretical models highlighting the importance of an individual’s actions in guiding their own evolutionary development are currently gaining more importance. In this respect, the recently proposed Relational Developmental Systems (RDS; Overton, 2015) metatheory collects those theories defending that change throughout the life cycle occurs through mutually influential relationships between individuals and their contexts. Thus, this perspective states that biological and environmental entities influence human development through a process of coaction. Specifically, Overton (2014) propose this metatheory based on the process-relational paradigm according to which any part of the developmental system is inextricably embodied by all other parts of the system. “Embodiment refers to the way individuals behave, experience, and live in the world by their being active agents with particular kinds of bodies; the body is integratively understood as form (a biological referent), as lived experience (a psychological referent), and as an entity in active engagement with the world (a sociocultural referent)” (Lerner, 2018, p. 25).

Therefore, attending to the importance of an individual’s actions in the relationship between body image and internalizing symptoms, it is imperative to understand the role that behaviors related to weight control and body image play when analyzing the different components of body image in adolescence. In this regard, BMI represents the physiological aspect of body changes during adolescence, body image perception (BIP) represents the cognitive component, and body image satisfaction (BIS) corresponds to the emotional component of body image. Moreover, these domains are also related to a behavioral component –dieting or exercising– that contributes to control weight and body image (Banfield and McCabe, 2002; Ramos et al., 2012).

Furthermore, some studies warn that these components –physiological, cognitive, emotional, and behavioral– interrelate differently depending on the adolescent’s sociodemographic factors [i.e., sex, age, and socioeconomic status (SES)]. Firstly, concern for body image differs according to sex; whereas boys tend to be more concerned with having a muscular body image, girls are more likely to have beauty ideals that are inseparable from thinness, in most cases below a healthy size (Neumark-Sztainer et al., 1999; Cafri and Thompson, 2004). In addition, although a higher proportion of girls tend to have a normal BMI compared to boys, they also show a greater prevalence of suffering misperceptions about their body image leading to a greater number of weight-control behaviors (Barker and Galambos, 2003; Larson et al., 2009; Ramos et al., 2013). Systematic literature reviews about sex inequalities in body dissatisfaction, such as those conducted by McCabe and Ricciardelli (2004) and Sobrino-Bazaga and Rabito-Alcoón (2018), support this perspective. However, there is less research regarding sex differences in the relationship between body image problems and IS in adolescence. Current studies such as that published by Bacopoulou et al. (2018) point out that girls showing higher anxiety on the internalizing subscale of the Youth Self-Report (YSR) have a higher risk of developing eating disorders than girls without anxiety, whereas this does not occur in their male peers.

Secondly, with regards to age researchers agree that both boys and girls are less satisfied with their body image throughout the period stretching from adolescence to adulthood (Sobrino-Bazaga and Rabito-Alcoón, 2018). However, during adolescence it appears that only girls decrease their body satisfaction with age (e.g., Bully and Elosua, 2011). This same tendency has been found in other variables such as BIP and weight control (Neumark-Sztainer et al., 1999; Barker and Galambos, 2003; McArthur et al., 2005). These results indicate that during adolescence girls’ association of ideal beauty with being thin, in most cases below a healthy size, is strengthened. However, there are no results that directly address how the relationship varies between body image and IS according to the age of the adolescents, and therefore remains an important unanswered question.

Thirdly, adolescents from families with a low SES tend to present higher rates of overweightness and obesity (O’Dea and Caputi, 2001; Cheung et al., 2011; Pereira et al., 2011). In addition, pertaining to families with a low SES also contributes to the persistence of meal-related health problems during this life stage (e.g., Wadsworth and Achenbach, 2005). Nevertheless, there is little research exploring the impact of SES on IS, or how SES moderates the association between body image and IS, and results are inconsistent. For example, in the study conducted by Ter Bogt et al. (2006) both boys and girls across all SES showed to experience elevated levels of IS when perceiving their body as too thin or (more frequently) too heavy. Another study (Zeller et al., 2004) conducted with 121 obese children and adolescents and their mothers found that in mothers with distress, low SES related to higher IS in their obese children. However, this study did not find a relationship between SES and IS self-reported by the children themselves.

In Spain, 17.2% of 11–18 year old adolescents show signs of being overweight or obese, with this percentage being clearly higher in boys and adolescents with a lower SES (Moreno et al., 2016). This study also shows that despite girls having a lower index of being overweight or obese, they are less satisfied with their body image and diet more frequently to lose weight, a behavior that also increases with age. Regarding internalizing symptoms, Oliva et al. (2015) underscores the higher proportion of internalizing symptoms in girls. In addition, unlike their male peers, in girls the internalizing symptoms increased with age and with a lower SES.

Therefore, in the context of the current research, it is important to further study the roles of sex, age, and socioeconomic level in the relationship between body image problems and IS in adolescence. With this objective we first propose to understand how the physiological (BMI), cognitive (BIP), and emotional (BIS) components of body image interrelate when predicting the adolescents’ internalizing symptoms. In addition, we will attempt to analyze the specific role that the variables sex, age, and socioeconomic level play in the aforementioned relationship. Along these lines, we assume that the exposure to double/triple/multiple factors of inequalities is more harmful than the exposure to a single one. Therefore, following an interesting approach from social sciences of exploring multiple inequalities and its intersectionality, mostly initiated from a gender perspective (Acker, 2006; Walby, 2011), this present study examined interactions between sex, age, and SES as sources of inequalities.

Secondly, this study proposes to further research those adolescents with worse scores in the specific component (physiological, cognitive, or emotional) that in the previous objective was the most important for IS. In this case, this study proposes to analyze in detail the profile of these adolescents, not only based on the physiological, cognitive, or emotional components, but also according to how the configuration of these variables relates with the behavioral component, measured through dieting and the frequency of physical activity.

## Materials and Methods

### Data

Data comes from the *Health Behaviour in School-aged Children* (HBSC) cross-sectional survey. The HBSC study is an international network supported by the World Health Organization that collects data in almost 50 countries in Europe and North America. The survey is conducted every 4 years with the aim of increasing knowledge about health-related behaviors, lifestyles, wellbeing, and developmental contexts of youth. Specifically, the data used in this paper is obtained from the 2018 edition of the HBSC study in Spain.

### Participants

This study is based on a nationally representative sample composed of 4531 adolescents aged 13–18 years who participated in the 2018 edition of the HBSC study in Spain. They were selected using random multi-stage sampling stratified by conglomerates according to age, type of school (public or private), and region (Moreno et al., 2018).

The sample was divided equally between boys and girls. The percentage of adolescents according to each age group was 16.7% who were 13–14, 51.3% were 15–16, and 32% were 17–18 years old. Likewise, the global sample was distributed between 63.3% that attended public schools, 31.7% that attended charter schools, and 5% that attended private schools. Regarding SES, 18.9% reported a low level, 48.8% a medium level, and 32.3% a high level.

Lastly, the sample was distributed between the different regions of Spain in the following percentages: 6.7% Andalucia, 4.1% Aragon, 4.6% Principado de Asturias, 7.2% Illes Balears, 5.5% Canarias, 3.7% Cantabria, 3.3% Castilla y León, 6.9% Castilla-La Mancha, 10.2% Cataluña, 6.6% Comunitat Valenciana, 5.7% Extremadura, 3.5% Galicia, 8.5% Comunidad de Madrid, 4.4% Región de Murcia, 6.4% Comunidad Foral de Navarra, 7.2% País Vasco, 3.6% La Rioja, and 1.9% Ceuta y Melilla.

### Instruments

The HBSC questionnaire is a broad survey that measures adolescents’ healthy habits, wellbeing, and their developmental contexts from a multidisciplinary perspective. The questionnaire uses a variety of validated scales and items. Spanish-language versions of the scales were used, that were translated/back-translated between English and Spanish following the guidelines and cooperation of the international coordination of the HBSC study (Roberts et al., 2009). The specific variables employed in this study –selected according to their scientific basis and study objectives– are detailed below.

Demographic variables were *sex* (students had to self-identify as a boy or girl) and SES evaluated using the Family Affluence Scale (FAS III, Schnohr et al., 2013). The FAS score was calculated as the sum of the responses from the six items included in the latest version of the instrument: “Does your family own a car, van or truck?” (0 = *no*; 1 = yes, one; 2 = *yes, two or more*); “Do you have your own bedroom for yourself?” (0 = *no*; 1 = *yes*); “How many computers do your family own (including laptops and tablets, not including game consoles and smartphones)?” (0 = *none*, 1 = *one*, 2 = two, 3 = *more than two*); “How many bathrooms (room with a bath/shower or both) are in your home?” (0 = *none*, 1 = *one*, 2 = *two*, 3 = *more than two*); “Does your family have a dishwasher at home?” (0 = no; 1 = yes); “How many times did you and your family travel out of Spain for a holiday/vacation last year?” (0 = *not at all*, 1 = *once*, 2 = *twice*, 3 = *more than twice*). The terciles (low, medium, and high), with cut-off points of 6 and 9 (in a range of 0–13) were used as a categorical variable.

This paper analyses different body-related variables:

– *Body Mass Index* is the ratio between self-reported weight and height (kg/m^2^). To use it as a categorical variable BMI was recoded according to the cut-off points proposed by Cole et al. (2000, 2007), with the options thin, normal, and overweight/obese.
– *Body Image Perception* was assessed with an item created for the HBSC study. Specifically, students were asked “Do you think your body is?” and their response options on a 5-point Likert scale ranged from 1 *much too thin*, to 5 *much too fat*. Responses were recoded into 3 categories: thin (1 *too thin*, and 2 *a bit thin*), normal (3 *I have the correct size*) and overweight (4 *a bit fat*, and 5 *too fat*).
– *Body Image Satisfaction* was assessed through the feelings and attitudes toward the body subscale of the Body Investment Scale (Orbach and Mikulincer, 1998). This subscale consists of 6 items (“I am frustrated with my physical appearance,” “I am satisfied with my appearance,” “I hate my body,” “I feel comfortable with my body,” “I feel anger toward my body,” and “I like my appearance in spite of its imperfections”). It is answered on a 5-point Likert scale from 1 *totally agree*, to 5 *totally disagree*. The Cronbach’s alpha in the analyzed sample was 0.89. In order to use it as a categorical variable, the sample was divided into terciles (low, medium, and high) with the cut-off points 3.6 and 4.5 (in a range of 1 to 5). When it is used as a continuous variable, high scores reflect high body satisfaction.

Two variables were included regarding the behavioral component: (1) a measure that was created by the international HBSC protocol to explore the behavior of dieting, asking adolescents whether they were currently dieting to lose or gain weight/volume; and (2) adolescents were asked about their level of Moderate to Vigorous Physical Activity (MVPA), as indicated by the number of days in which they felt physically active during a total of at least 60 min a day over the last 7 days. The response options ranged from 0 to 7 days (Prochaska et al., 2001). In addition, their level of Vigorous Physical Activity (VFA) was assessed as the frequency with which they engaged in some type of physical activity that made them sweat or out of breath in their free time outside of school hours. The response options on a Likert scale ranged from 1 *never*, to 7 *every day*.

Lastly, the variables related to internalizing mental health problems were assessed using the licensed version of the Spanish version of the YSR (Achenbach and Rescorla, 2001). The three internalizing syndrome scales employed were: anxious/depressed (13 items, with a 0 to 13 response range), withdrawn/depressed (8 items, with a 0 to 8 response range), and somatic complaints (10 items, with a 0 to 10 response range). All the items are Likert-type response format comprising three categories (0 = *not true*, 1 = *somewhat true or sometimes true*, 2 = *very true or often true*) and refer to symptoms and experiences from the past 6 months. The application guidelines marked by Achenbach (2013) were followed in the application of this instrument within the HBSC study in Spain. The Cronbach’s alpha in the present study was 0.79 for anxious/depressed, 0.75 for withdrawn/depressed, 0.76 for somatic complaints, and 0.87 for the global scale of IS. In order to use IS as categorical (for the second objective of the study) low (12–24), medium (24.50–32) and high (32.50–80) terciles were used. When it is used as a continuous variable, high scores reflect high internalizing symptoms.

### Procedure

The international coordination of the HBSC study stipulates three basic conditions that must be complied with during data collection: the questionnaires must be answered by the students themselves; the anonymity of the answers must be strictly guaranteed; and the questionnaires must be administered within the school context (Inchley et al., 2016). Thus, the data was always collected in the school setting under the supervision of teachers. In all the schools, instructions were given to the teachers who would be supervising the classroom when the adolescents responded to the questionnaires.

New information and communication technologies (ICT) based on a CAWI (Computer-Assisted Web Interviewing) model were used in data collection. The guided computerized procedure has the advantage of immediately receiving and incorporating the students’ responses in the database, thus reducing possible errors from the data entry process, as well as helping to maintain the anonymity of the responses. In addition, instructions for the students were included at the beginning of the questionnaire to guarantee homogeneity amongst all the participants.

In all the participating education centers, permission was requested from the committee board of each school and they handled the request of permission of the adolescents and their families according to each centers’ protocols. This study was approved by the Ethical Research Committee of the regional government of Andalusia, Spain (Junta de Andalucía).

Sample selection was carried out between February and June 2018. Once data were collected, they were cleaned and controlled according to the international HBSC protocols offered by the Norwegian Centre for Research Data (Currie et al., 2014). In this procedure, from the initial sample composed of 5356 adolescents, subjects who showed a response rate below 40% of the total questionnaire (9.5% of the initial sample), who did not give basic sociodemographic information (0.9% of the initial sample), who showed an overly elevated pattern in their answers (4.3% of the initial sample), or who had not responded to all the analyzed variables (3.5% of the initial sample) were eliminated. Due to the large remaining sample (4531 adolescents), the decision made was to not impute the missing values and instead analyze only those subjects with valid values in the analyzed variables.

### Statistical Analysis

Bivariate analyses including chi-square, *t*-test and ANOVA were used, requiring a significance level of 0.01. The corresponding effect size was reported in every case. Thus, *phi*, Crammer’s *V* and *Tau-b* were calculated to determine the effect size in the comparison of proportions, with the following cut-off points: 0–0.09 = negligible, 0.10–0.29 = small, 0.30–0.49 = medium, 0.50 and above = high. Cohen’s *d* was calculated in the comparison of two groups’ means, with the following cut-off points: 0–0.19 = negligible, 0.20–0.49 = small, 0.50–0.79 = medium, 0.80 and above = high. Eta-squared was calculated in the comparison of more than two groups’ means, with the following cut-off points: 0–0.009 = negligible, 0.010–0.059 = small, 0.060–0.139 = medium, 0.140 and above = high. Pearson’s correlation was calculated in the relationship between two quantitative variables, with cut-off points 0–0.090 = negligible, 0.10–0.239 = small, 0.24–0.369 = medium, 0.37 and above = high (Cohen, 1988).

Linear regression analysis was used to examine the relationship between sociodemographic variables (sex, age, and FAS) and the indicators of body image on the total scale of IS and each one of the subscales (anxious/depressed, withdrawn/depressed, and somatic complaints). The principal effects were analyzed first, and afterward the previous models were replicated adding the interaction effects. Pearson’s correlations were analyzed to determine the significant interaction effects, segmented according to the variables implicated in said interaction. Differences in correlations were compared using the Fisher’s *Z*-test, establishing the cut-off point in values above 2.33 (in absolute values), corresponding to a 99% unilateral confidence interval.

To analyze the characteristics that define the two groups established for the second objective of the present study, a logistical regression model was created which incorporated the value of the odds ratio with its corresponding confidence interval.

Statistical analyses were conducted using the IBM SPSS Statistics 25.0 software.

## Results

### Analysis of the Bivariate Relationship Between Variables

Firstly, the distribution of the sample in the three components of body image (BMI, BIP, and BIS) is shown in Table 1. These variables presented a high correlation between them. Specifically, a higher BMI was positively related with overweight BIP (*r* = 0.514, *p* < 0.001) and negatively associated with BIS (*r* = −0.268, *p* < 0.001). Psychological components (BIP and BIS) were also negatively correlated, indicating that overweight BIP is associated with a lower BIS (*r* = −0.410, *p* < 0.001).

**TABLE 1:** Sample description in the three variables related to body image, dieting, and physical activity.

		***N***	**Percentage**
BMI	Thin	617	13.6%
	Normal	3197	70.6%
	Overweight/obese	717	15.8%
BIP	Thin	803	17.7%
	Normal	2195	48.4%
	Overweight	1533	33.8%
BIS	Low	1625	35.9%
	Medium	1673	36.9%
	High	1233	27.2%
Diet to lose	No	3941	87.2%
	Yes	581	12.8%
Diet to gain	No	5098	96.2%
	Yes	200	3.8%
MVPA	Null	316	7.4%
	Low	2739	64.5%
	High	1191	28.0%
VPA	Null	527	12.4%
	Low	2319	54.6%
	High	1405	33.1%

*BMI, Body Mass Index; BIP, Body Image Perception; BIS, Body Image Satisfaction; MVPA, Moderate-Vigorous Physical Activity; VPA, Vigorous Physical Activity.*

These three components of body image varied according to the adolescents’ sex, age, and socioeconomic level. As shown in Table 2, BMI was higher in 15–16 year old adolescents compared to the 13–14 year olds, as well as being higher in adolescents with a low FAS score. Regarding overweight BIP, this was higher in girls and in adolescents with low FAS. Lastly, BIS was lower in girls, 17–18 year old adolescents, and those with low FAS.

**TABLE 2:** Means (standard deviations), significance test and effect sizes of sex, age, family affluence, dieting, and physical activity with the three variables related with body image.

		**Body Mass Index**		**Body Image Perception**		**Body Image Satisfaction**	
				
		**Descriptive statistics**	**Significance tests**	**Descriptive statistics**	**Significance tests**	**Descriptive statistics**	**Significance tests**
Sex	Boys	21.38 (3.93)	*t*(4529) = 4.08,	3.04 (0.81)	*t*(4529) = 11.10,	4.13 (0.80)	*t*(4529) = 15.53,
	Girls	20.93 (3.47)	*p* < *0.001*; *d* = 0.12	3.31 (0.83)	*p* < *0.001*; *d* = **0.33**	3.72 (0.96)	*p* < 0.001; *d* = **0.46**
Age	13–14 year	20.31 (3.55)	*F*(4528) = 36.99,	3.16 (0.79)	*F*(4528) = 0.30,	4.04 (0.88)	*F*(4528) = 10.76,
	15–16 year	21.08 (3.61)	*p* < 0.001; η^2^ = 0.02	3.17 (0.82)	*p* = 0.74; η^2^ < 0.01	3.94 (0.91)	*p* < 0.001; η^2^ = 0.01
	17–18 year	21.71 (3.87)	*d* = **0.21**/0.17/**0.37**	3.19 (0.88)	*d* = 0.01/0.02/0.03	3.85 (0.92)	*d* = 0.11/0.10/**0.21**
FAS	Low	21.92 (4.48)	*F*(4528) = 29.57,	3.27 (0.90)	*F*(4528) = 9.70,	3.77 (0.98)	*F*(4528) = 18.66,
	Medium	21.19 (3.58)	*p* < 0.001; η^2^ = 0.01	3.18 (0.82)	*p* < 0.001; η^2^ = 0.01	3.94 (0.90)	*p* < 0.001; η^2^ = 0.01
	High	20.66 (3.20)	*d* = 0.19/0.15/**0.34**	3.11 (0.80)	*d* = 0.11/0.09/**0.20**	4.02 (0.87)	*d* = 0.18/0.09/**0.26**
Diet to lose	No	20.75 (3.38)	*t*(4765) = −19.87,	3.07 (0.80)	*t*(5233) = 23.78,	4.02 (0.85)	*t*(5007) = 1.96,
	Yes	23.90 (4.65)	*p* < *0.001*; *d* = **0.88**	3.90 (0.68)	*p* < *0.001*; *d* = **1.05**	3.27 (1.03)	*p* < *0.001*; *d* = **0.86**
Diet to gain	No	21.21 (3.76)	*t*(4765) = 4.07,	3.22 (0.83)	*t*(5233) = 13.33,	3.90 (0.93)	*t*(5007) = 1.96,
	Yes	20.07 (3.05)	*p* < *0.001*; *d* = **0.30**	2.41 (0.83)	*p* < *0.001*; *d* = **0.98**	4.04 (0.84)	*p* = 0.050; *d* = 0.15
MVPA	Null	21.81 (4.79)	*F*(4528) = 18.20,	3.35 (1.00)	*F*(4528) = 28.55,	3.47 (1.08)	*F*(4528) = 157.99,
	Low	21.31 (3.67)	*p* < 0.001; η^2^ = 0.01	3.22 (0.85)	*p* < 0.001; η^2^ = 0.01	3.83 (0.90)	*p* < 0.001; η^2^ = 0.07
	High	20.65 (3.43)	*d* = 0.13/0.18/**0.30**	3.03 (0.69)	*d* = 0.15/**0.24/0.42**	4.28 (0.74)	*d* = **0.39/0.53/0.98**
VPA	Null	21.25 (4.73)	*F*(4528) = 0.60,	3.19 (0.95)	*F*(4528) = 9.13,	3.64 (1.04)	*F*(4528) = 74.10,
	Low	21.20 (3.74)	*p* = 0.55; η^2^ < 0.01	3.22 (0.84)	*p* < 0.001; η^2^ < 0.01	3.87 (0.90)	*p* < 0.001; η^2^ = 0.03
	High	21.08 (3.26)	*d* = 0.01/0.03/0.05	3.04 (0.81)	*d* = 0.03/**0.22**/0.18	4.14 (0.82)	*d* = **0.25/0.31/0.56**

*FAS, Family Affluence Scale; MVPA, Moderate-Vigorous Physical Activity; VPA, Vigorous Physical Activity; y, years-old. In contrasts of d of Cohen with variables of three values: the d in first position corresponds to the contrast of the 1st and 2nd value, the d in second position corresponds to the contrast of the 2nd and 3rd value and the d in third position corresponds to the contrast of the 1st and 3rd value. The bold values indicate small (0.20–0.49), medium (0.50–0.79), or high (0.80 and above) effect size values.*

Differences were also found in these three components of body image in their relationship with indicators of IS, as shown in Table 3. Specifically, the adolescents with higher BMI showed higher scores in withdrawn/depressed and in the global scale of IS. However, a relationship was found between all the indicators of IS and BIS and BIP: those adolescents with overweight BIP and low BIS were more anxious/depressed, withdrawn/depressed, had more somatic complaints, and had a higher global IS score.

**TABLE 3:** Means (standard deviations), significance test and effect sizes of the three variables related with body image, sex, age, family affluence, dieting, and physical activity with anxious/depressed, withdrawn/depressed, somatic complaints, and total of internalizing symptoms.

		**Anxious/depressed**		**Withdrawn/depressed**		**Somatic complaints**		**Internalizing symptoms (total)**	
					
		**Descriptive s.**	**Significance tests**	**Descriptive s.**	**Significance tests**	**Descriptive s.**	**Significance tests**	**Descriptive s.**	**Significance tests**
BMI	Thin	6.34 (4.01)	*F*(4777) = 7.59,	3.60 (2.81)	*F*(4777) = 16.68,	3.61 (3.12)	*F*(4777) = 5.83,	13.77 (10.56)	*F*(4777) = 48.97,
	Normal	6.05 (4.07)	*p* = 0.001; η^2^ < 0.01	3.65 (2.91)	*p* < 0.001; η^2^ < 0.01	3.49 (3.05)	*p* = 0.003; η^2^ < 0.01	17.00 (9.60)	*p* < 0.001; η^2^ = 0.02
	Overweight/obese	6.65 (4.43)	*d* = 0.07/0.15/0.08	4.31 (3.12)	*d* = 0.01/**0.22/0.23**	3.90 (3.40)	*d* = 0.05/0.14/0.09	18.97 (10.28)	*d* = **0.33/0.23/0.72**
BIP	Thin	6.25 (4.00)	*F*(5248) = 162.23,	3.81 (2.97)	*F*(5248) = 135.79,	3.49 (2.94)	*F*(5248) = 114.44,	16.9 (9.61)	*F*(5248) = 136.94,
	Normal	5.24 (3.57)	*p* < 0.001; η^2^ = 0.06	3.10 (2.62)	*p* < 0.001; η^2^ = 0.05	2.97 (2.75)	*p* < 0.001; η^2^ = 0.04	14.71 (8.73)	*p* < 0.001; η^2^ = 0.05
	Overweight	7.50 (4.55)	*d* = **0.29/0.56/0.27**	4.64 (3.12)	*d* = 0.15/**0.31/0.22**	4.48 (3.48)	*d* = 0.19/**0.46/0.27**	19.96 (10.90)	*d* = **0.33/0.20/0.49**
BIS	Low	8.15 (4.63)	*F*(5022) = 402.48,	5.01 (3.16)	*F*(5022) = 311.59,	4.71 (3.61)	*F*(5022) = 221.54,	21.49 (11.00)	*F*(5022) = 375.15,
	Medium	5.53 (3.40)	*p* < 0.001; η^2^ = 0.14	3.38 (2.61)	*p* < 0.001; η^2^ = 0.11	3.17 (2.64)	*p* < 0.001; η^2^ = 0.08	15.42 (8.35)	*p* < 0.001; η^2^ = 0.13
	High	4.48 (3.20)	*d* = **0.66/0.30/0.89**	2.59 (2.41)	*d* = **0.56/0.29/0.83**	2.63 (2.53)	*d* = **0.51**/0.19/**0.65**	12.76 (7.87)	*d* = **0.64/0.29/0.87**
Sex	Boys	5.09 (3.68)	*t*(5410) = −19.11,	3.44 (2.86)	*t*(5410) = −7.37,	2.74 (2.66)	*t*(5410) = −19.36,	14.77 (8.77)	*t*(5410) = −14.57,
	Girls	7.28 (4.26)	*p* < *0.001*; *d* = **0.52**	4.06 (2.99)	*p* < *0.001*; *d* = **0.20**	4.41 (3.31)	*p* < *0.001*; *d* = **0.53**	18.98 (10.59)	*p* < *0.001*; *d* = **0.40**
Age	13–14 year	5.97 (3.90)	*F*(5410) = 2.44,	3.23 (2.75)	*F*(5410) = 18.10,	3.51 (3.03)	*F*(5410) = 7.55,	14.24 (10.03)	*F*(5410) = 47.61,
	15–16 year	6.13 (4.17)	*p* = 0.087; η^2^ < 0.01	3.72 (2.90)	*p* < 0.001; η^2^ < 0.01	3.43 (3.06)	*p* = 0.001; η^2^ < 0.01	16.85 (9.63)	*p* < 0.001; η^2^ = 0.02
	17–18 year	6.38 (4.17)	*d* = 0.03/0.05/0.08	4.07 (3.06)	*d* = 0.15/0.10/**0.24**	3.83 (3.24)	*d* = 0.02/0.12/0.10	18.29 (10.13)	*d* = **0.28**/0.12/**0.39**
FAS	Low	6.73 (4.33)	*F*(4783) = 12.15,	4.31 (2.99)	*F*(4783) = 27.04,	4.15 (3.36)	*F*(4783) = 18.41,	18.04 (10.79)	*F*(4783) = 7.77,
	Medium	6.11 (4.09)	*p* < 0.001; η^2^ < 0.01	3.76 (2.93)	*p* < 0.001; η^2^ = 0.01	3.4 (2.91)	*p* < 0.001; η^2^ < 0.01	16.69 (9.69)	*p* < 0.001; η^2^ < 0.01
	High	5.83 (3.95)	*d* = 0.13/0.07/**0.21**	3.35 (2.81)	*d* = 0.19/0.15/**0.34**	3.42 (3.16)	*d* = **0.20**/0.05/**0.21**	16.35 (9.44)	*d* = 0.12/0.08/0.14
Diet to lose	No	5.99 (3.99)	*t*(5297) = −8.38,	3.66 (2.90)	*t*(5297) = −5.37,	3.42 (3.01)	*t*(5297) = −8.61,	16.55 (9.64)	*t*(5297) = −5.72,
	Yes	7.52 (4.77)	*p* < *0.001*; *d* = **0.37**	4.36 (3.13)	*p* < *0.001*; *d* = **0.22**	4.61 (3.66)	*p* < *0.001*; *d* = **0.36**	19.07 (11.59)	*p* < *0.001*; *d* = **0.26**
Diet to gain	No	6.20 (4.12)	*t*(5297) = 0.19,	3.74 (2.93)	*t*(5297) = −0.81,	3.57 (3.11)	*t*(5297) = −0.94,	16.84 (9.95)	*t*(5297) = −1.96,
	Yes	5.97 (4.37)	*p* = 0.058; *d* = 0.01	3.90 (3.27)	*p* = 0.417; *d* = 0.06	3.73 (3.49)	*p* = 0.348; *d* = 0.07	17.90 (9.99)	*p* = 0.051; *d* = 0.14
MVPA	Null	7.74 (4.92)	*F*(4902) = 94.18,	5.17 (3.52)	*F*(4902) = 154.33,	4.68 (3.73)	*F*(4902) = 95.49,	21.96 (11.55)	*F*(4902) = 169.97,
	Low	6.49 (4.08)	*p* < 0.001; η^2^ = 0.04	4.04 (2.92)	*p* < 0.001; η^2^ = 0.06	3.80 (3.13)	*p* < 0.001; η^2^ = 0.04	17.89 (9.69)	*p* < 0.001; η^2^ = 0.07
	High	5.00 (3.64)	*d* = **0.35/0.35/0.71**	2.67 (2.40)	*d* = **0.43/0.46/0.93**	2.68 (2.6)	*d* = **0.33/0.36/0.72**	13.05 (8.46)	*d* = **0.44/0.48/0.95**
VPA	Null	7.65 (4.83)	*F*(4909) = 66.01,	5.05 (3.38)	*F*(4909) = 97.22,	4.23 (3.41)	*F*(4909) = 49.70,	20.15 (11.56)	*F*(4909) = 58.70,
	Low	6.31 (3.99)	*p* < 0.001; η^2^ = 0.03	3.80 (2.82)	*p* < 0.001; η^2^ = 0.04	3.77 (3.12)	*p* < 0.001; η^2^ = 0.02	17.17 (9.66)	*p* < 0.001; η^2^ = 0.02
	High	5.35 (3.79)	*d* = **0.33/0.21/0.53**	3.13 (2.73)	*d* = **0.44/0.21/0.64**	2.92 (2.82)	*d* = 0.17/**0.25/0.42**	15.00 (9.04)	*d* = **0.32/0.29/0.50**

*BMI, Body Mass Index; BIP, Body Image Perception; BIS, Body Image Satisfaction; FAS, Family Affluence Scale; MVPA, Moderate-Vigorous Physical Activity; VPA, Vigorous Physical Activity; y, years-old; s, statistics. In contrasts of d of Cohen with variables of three values: the d in first position corresponds to the contrast of the 1st and 2nd value, the d in second position corresponds to the contrast of the 2nd and 3rd value and the d in third position corresponds to the contrast of the 1st and 3rd value. The bold values indicate small (0.20–0.49), medium (0.50–0.79), or high (0.80 and above) effect size values.*

Analysis of IS distribution according to sociodemographic variables (shown in Table 3) also revealed inequalities according to sex (girls had higher scores in all indicators analyzed), age (differences were lower than in sex, however older adolescents had higher scores in the withdrawn/depressed subscale and in the global IS score), and FAS (adolescents with lower family affluence scored higher in the three subscales, but the global score did not show relevant differences).

Examining behaviors related to weight control and body image, Table 1 shows the distribution of the sample in these variables. Table 3 shows no relationship with the behavior of gaining weight/volume but does with the weight-loss diets. Specifically, adolescents that dieted to lose weight/volume had higher scores in all the subscales and in the global scale of IS. This same relationship was found with the two indicators of physical activity, meaning that those adolescents who did less physical activity (either MVPA or VPA) showed higher scores in all the internalizing indicators.

Returning to the body image variables, Table 2 also shows the bivariate relationship between the three components of body image and the weight control and body image behaviors. In general, adolescents with higher BMI more frequently dieted to lose weight/volume and did MVPA. Adolescents with an overweight BIP showed a higher frequency of dieting to lose weight/volume, and a lower tendency to practice either type of physical activity, as well as a lower frequency of dieting to gain weight/volume. Those adolescents with lower BIS dieted more to lose weight/volume and were more inclined to not practice either of the two types of physical activity (MVPA or VPA).

Regarding the relationship between these weight and body image behaviors, differences were found according to sex, age, and FAS score, as shown in Table 4. Specifically, dieting to lose weight/volume was more frequent in girls. On the contrary, dieting to gain weight/volume was more frequent in boys, those higher in age, and with high FAS. On the other hand, both MVPA and VPA were more frequent in boys, of a lower age, and with high FAS.

**TABLE 4:** Percentage, significance test and effect sizes of sex, age, and family affluence with dieting and physical activity.

		**Diet to lose**			**Diet to gain**			**MVPA**				**VPA**			
					
		**Descriptive**		**Significance tests**	**Descriptive**		**Significance tests**	**Descriptive**			**Significance tests**	**Descriptive**			**Significance tests**
									
		**statistics**			**statistics**			**statistics**				**statistics**			
		**No**	**Yes**		**No**	**Yes**		**Null**	**Low**	**High**		**Null**	**Low**	**High**	
Sex	Boys	90.0%	10.0%	*X*^2^(1) = 32.73, *p* < 0.001; *phi* = 0.085	94.2%	5.8%	*X*^2^(1) = 45.57, *p* < 0.001; *phi* = **0.100**	5.3%	58.1%	36.6%	*X*^2^(2) = 161.71, *p* < 0.001; *V* = **0.195**	8.1%	47.1%	44.8%	*X*^2^(2) = 281.65, *p* < 0.001; *V* = **0.257**
		**(5.7)**	**(−5.7)**		**(−6.8)**	**(6.8)**		**(−5.2)**	**(−8.7)**	**(12.3)**		**(−8.5)**	**(−9.6)**	**(16.2)**	
	Girls	84.3%	15.7%		98.1%	1.9%		9.5%	70.9%	19.6%		16.7%	61.9%	21.4%	
		**(−5.7)**	**(5.7)**		**(6.8)**	**(−6.8)**		**(5.2)**	**(8.7)**	**(−12.3)**		**(8.5)**	**(9.6)**	**(−16.2)**	
Age	13–14 year	87.3%	12.7%	*X*^2^(2) = 0.82, *p* = 0.664; *V* = 0.013	97.7%	2.3%	*X*^2^(2) = 19.77, *p* < 0.001; *V* = 0.066	4.3%	61.3%	34.4%	*X*^2^(4) = 62.72, *p* < 0.001; *Tau -b* = −0.106	8.4%	55.9%	35.7%	*X*^2^(4) = 31.02, *p* < 0.001; *Tau -b* = −0.070
		(0.1)	(−0.1)		**(2.5)**	**(−2.5)**		**(−3.5)**	(−2.0)	**(4.1)**		**(−3.6)**	(0.8)	(1.6)	
	15–16 year	87.5%	12.5%		96.8%	3.2%		6.3%	64.3%	29.4%		12.0%	53.1%	34.9%	
		(0.7)	(−0.7)		(2.1)	(−2.1)		**(−2.9)**	(−0.3)	(2.0)		(−0.8)	(−2.0)	**(2.6)**	
	17–18 year	86.5%	13.5%		94.4%	5.6%		10.9%	66.5%	22.6%		15.2%	56.2%	28.7%	
		(−0.9)	(0.9)		**(−4.3)**	**(4.3)**		**(5.9)**	(1.9)	**(−5.4)**		**(3.7)**	(1.4)	**(−4.1)**	
FAS	Low	86.7%	13.3%	*X*^2^(2) = 0.22, *p* = 0.894; *V* = 0.007	96.6%	3.4%	*X*^2^(2) = 6.81, *p* = 0.033; *V* = 0.041	10.0%	66.4%	23.7%	*X*^2^(4) = 28.58, *p* < 0.001; *Tau -b* = 0.075	17.0%	56.3%	26.7%	*X*^2^(4) = 47.29, *p* < 0.001; *Tau -b* = 0.093
		(−0.5)	(0.5)		(0.7)	(−0.7)		**(3.1)**	(1.2)	**(−3.1)**		**(4.4)**	(1.0)	**(−4.1)**	
	Medium	87.4%	12.6%		96.7%	3.3%		7.3%	65.7%	27.0%		11.9%	56.6%	31.5%	
		(0.2)	(−0.2)		(1.9)	(−1.9)		(−0.1)	(1.5)	(−1.5)		(−0.8)	(2.3)	(−1.8)	
	High	87.3%	12.7%		95.0%	5.0%		5.9%	61.8%	32.4%		10.2%	51.1%	38.7%	
		(0.1)	(−0.1)		**(−2.6)**	**(2.6)**		**(−2.5)**	**(−2.6)**	**(4.2)**		**(−2.8)**	**(−3.3)**	**(5.4)**	

*The value of the corrected standardized residual is included in parentheses. The bold values indicate small (0.10–0.29), medium (0.30–0.49), or high (0.50 and above) effect size values- phi and V- and corrected standardized residuals -SRc- higher to 2.33 (one-sided confidence interval of 99%).*

### The Predictive Capacity of Body Image Variables (BMI, BIP, and BIS) on IS, Considering the Influence of Sociodemographic Variables (Sex, Age, and Family Affluence)

Table 5 shows the results of the linear regression analyses conducted for each of the four measures of IS (anxious/depressed, withdrawn/depressed, and somatic complaints) and for the global scale of IS. Two types of linear regression analyses were conducted for each internalizing indicator: one simple and another global. Whereas the simple analysis only measured the direct influence of the independent variables, the global analysis controlled possible interaction effects of the sociodemographic variables in the relationship between body image and the internalizing indicators.

**TABLE 5:** Linear regression analysis of the simple model, only with main effects, and the global model, with interaction effects, on anxious/depressed, withdrawn/depressed, somatic complaints, and total of internalizing symptoms.

	**Anxious/depressed**	**Withdrawn/depressed**	**Somatic complaints**	**Internalizing symptoms (total)**
**Simple model (without interaction effects)**				
**R Squared**	**0.245**	**0.172**	**0.154**	**0.228**
Sex	$\eta_{P}^{2}$ = 0.037; *p* < 0.001	$\eta_{P}^{2}$ < 0.001; *p* = 0.173	$\eta_{P}^{2}$ = 0.045; *p* < 0.001	$\eta_{P}^{2}$ = 0.024; *p* < 0.001
Age	$\eta_{P}^{2}$ < 0.001; *p* = 0.850	$\eta_{P}^{2}$ = 0.005; *p* < 0.001	$\eta_{P}^{2}$ < 0.001; *p* = 0.309	$\eta_{P}^{2}$ = 0.013; *p* < 0.001
FAS	$\eta_{P}^{2}$ = 0.003; *p* = 0.001	$\eta_{P}^{2}$ = 0.009; *p* < 0.001	$\eta_{P}^{2}$ = 0.003; *p* = 0.001	$\eta_{P}^{2}$ < 0.001; *p* = *0.375*
BMI	$\eta_{P}^{2}$ < 0.001; *p* = 0.182	$\eta_{P}^{2}$ < 0.001; *p* = 0.311	$\eta_{P}^{2}$ < 0.001; *p* = 0.878	$\eta_{P}^{2}$ = 0.012; *p* < 0.001
BIP	$\eta_{P}^{2}$ = 0.001; *p* = 0.017	$\eta_{P}^{2}$ = 0.001; *p* = 0.025	$\eta_{P}^{2}$ < 0.001; *p* = 0.961	$\eta_{P}^{2}$ = 0.007; *p* < 0.001
BIS	$\eta_{P}^{2}$ = 0.166; *p* < 0.001	$\eta_{P}^{2}$ = 0.127; *p* < 0.001	$\eta_{P}^{2}$ = 0.068; *p* < 0.001	$\eta_{P}^{2}$ = 0.140; *p* < 0.001
**Global model (with interaction effects)**				
**R Squared**	**0.253**	**0.174**	**0.163**	**0.235**
**Main effects**				
Sex	$\eta_{P}^{2}$ = 0.005; *p* < 0.001	$\eta_{P}^{2}$ < 0.001; *p* = 0.573	$\eta_{P}^{2}$ < 0.001; *p* = 0.246	$\eta_{P}^{2}$ = 0.006; *p* < 0.001
Age	$\eta_{P}^{2}$ = 0.001; *p* = 0.016	$\eta_{P}^{2}$ = 0.005; *p* < 0.001	$\eta_{P}^{2}$ < 0.001; *p* = 0.483	$\eta_{P}^{2}$ = 0.003; *p* < 0.001
FAS	$\eta_{P}^{2}$ = 0.001; *p* = 0.103	$\eta_{P}^{2}$ < 0.001; *p* = 0.635	$\eta_{P}^{2}$ = 0.002; *p* = 0.002	$\eta_{P}^{2}$ < 0.001; *p* = *0.929*
BMI	$\eta_{P}^{2}$ = 0.004; *p* < 0.001	$\eta_{P}^{2}$ < 0.001; *p* = 0.389	$\eta_{P}^{2}$ = 0.001; *p* = 0.016	$\eta_{P}^{2}$ = 0.002; *p* = *0.001*
BIP	$\eta_{P}^{2}$ < 0.001; *p* = 0.852	$\eta_{P}^{2}$ < 0.001; *p* = 0.511	$\eta_{P}^{2}$ < 0.001; *p* = 0.374	$\eta_{P}^{2}$ = 0.002; *p* = *0.007*
BIS	$\eta_{P}^{2}$ = 0.002; *p* = 0.004	$\eta_{P}^{2}$ = 0.012; *p* < 0.001	$\eta_{P}^{2}$ = 0.002; *p* = 0.001	$\eta_{P}^{2}$ = 0.010; *p* < 0.001
**Interaction 1st order effects**				
Sex x BMI	n.s.	n.s.	$\eta_{P}^{2}$ = 0.002; *p* = *0.002*	n.s.
Sex x BIP	$\eta_{P}^{2}$ = 0.002; *p* = 0.004	$\eta_{P}^{2}$ = 0.002; *p* = 0.003	$\eta_{P}^{2}$ = 0.002; *p* = 0.003	$\eta_{P}^{2}$ = 0.003; *p* < 0.001
Sex x BIS	n.s.	$\eta_{P}^{2}$ = 0.003; *p* = 0.001	n.s.	n.s.
Age x BMI	$\eta_{P}^{2}$ = 0.004; *p* < 0.001	n.s.	n.s.	$\eta_{P}^{2}$ = 0.002; *p* = *0.007*
Age x BIP	n.s.	n.s.	n.s.	n.s.
Age x BIS	n.s.	n.s.	n.s.	n.s.
FAS x BMI	$\eta_{P}^{2}$ = 0.002; *p* = 0.005	n.s.	n.s.	n.s.
FAS x BIP	n.s.	n.s.	n.s.	n.s.
FAS x BIS	n.s.	n.s.	n.s.	n.s.
**Interaction 2nd order effects**				
(Sex x Age) x BMI	n.s.	n.s.	$\eta_{P}^{2}$ = 0.003; *p* = 0.001	n.s.
(Sex x Age) x BIP	n.s.	n.s.	n.s.	n.s.
(Sex x Age) x BIS	n.s.	n.s.	n.s.	n.s.
(Sex x FAS) x BMI	n.s.	n.s.	n.s.	n.s.
(Sex x FAS) x BIP	$\eta_{P}^{2}$ = 0.003; *p* = 0.001	$\eta_{P}^{2}$ = 0.003; *p* = 0.005	n.s.	$\eta_{P}^{2}$ = 0.005; *p* < 0.001
(Sex x FAS) x BIS	$\eta_{P}^{2}$ = 0.004; *p* < 0.001	$\eta_{P}^{2}$ = 0.003; *p* = 0.001	$\eta_{P}^{2}$ = 0.003; *p* = *0.001*	$\eta_{P}^{2}$ = 0.008; *p* < 0.001
(Age x FAS) x BMI	n.s.	n.s.	n.s.	n.s.
(Age x FAS) x BIP	n.s.	n.s.	n.s.	n.s.
(Age x FAS) x BIS	n.s.	n.s.	n.s.	n.s.

*FAS, Family Affluence Scale; BMI, Body Mass Index; BIP, Body Image Perception; BIS, Body Image Satisfaction; n.s., not significant.*

Regarding the individual predictive capacity (without interaction) of the sociodemographic and body image variables on the four internalizing indicators, results showed BIS as the variable with the most weight. Therefore, BIS showed to be the most important predictor of IS, whether in the global score ($\eta_{P}^{2}=0.140$) or in the three subscales: anxious/depressed ($\eta_{P}^{2}=0.166$), withdrawn/depressed ($\eta_{P}^{2}=0.127$), and somatic complaints ($\eta_{P}^{2}=0.068$). The adolescents’ sex was the second most important dependent variable, both in the global scale and in the anxious/depressed and withdrawn/depressed scales. Age was the second highest predictive variable of withdrawn/depressed and the third in the global scale.

Following this initial linear regression analysis, interaction of the sociodemographic variables was included both independently –as shown in Table 6– as well as simultaneously combining two of these demographic variables –as shown in Table 7. This exhaustive control of demographic variables increased the percentages of explained variance of the four regression analyses. Thus, the global scale showed a 23.5% predictive capacity, 25.3% for anxious/depressed, 17.4% for withdrawn/depressed, and 16.3% for somatic complaints. In addition, congruent with the aforementioned, the main effects of these global linear regression models once again demonstrated the importance of BIS. Specifically, BIS is the only dependent variable amongst the first three positions in the prediction of all the dependent variables, that is to say, both in the global scale and the three subscales.

**TABLE 6:** Pearson’s correlation and Fisher’s *Z* between variables of first-order interaction effects of the linear regression analysis shown in Table 5.

	**Anxious/depressed**	**Withdrawn/depressed**	**Somatic complaints**	**Internalizing symptoms (total)**
**Correlation according to sex**	**Boys/Girls**	**Boys/Girls**	**Boys/Girls**	**Boys/Girls**

BMI	–	–	0.041/0.138^∗^	–
			*Z* = **3.38**	
BIP	0.068^∗^/0.179^∗^	0.071^∗^/0.165^∗^	0.046/0.170^∗^	0.044/0.177^∗^
BIP	*Z* = **4.09**	Z = **3.45**	*Z* = **4.55**	*Z* = **4.88**
BIS		−0.350^∗^/−0.411^∗^	–	–
		*Z* = **2.53**		

**Correlation according to age**	**13–14/15–16/17–18**	**13–14/15–16/17–18**	**13–14/15–16/17–18**	**13–14/15–16/17–18**

BMI	0.207^∗^/0.051/0.045	–	–	0.274^∗^/0.147/0.164
	Z_(13__–__14 to 15__–__16)_ = **4.43**			Z_(13__–__14 to 15__–__16)_ = **3.27**
	Z_(15__–__16 to 17__–__18)_ = 0.18			Z_(15__–__16 to 17__–__18)_ = 0.53
	Z_(13__–__14 to 17__–__18)_ = **3.78**			Z_(13__–__14 to 17__–__18)_ = **2.65**
BIP	–	–	–	–
BIS	–	–	–	–

**Correlation according to FAS**	**Low/Medium/High**	**Low/Medium/High**	**Low/Medium/High**	**Low/Medium/High**

BMI	0.164^∗^/0.049/0.019	–	–	
	*Z*_(__L__–__M)_ = **2.83**			
	Z_(M__–__H)_ = 1.54			
	*Z*_(L__–__H)_ = **3.53**			
BIP	–	–	–	–
BIS	–	–	–	–

*FAS, Family Affluence Scale; BMI, Body Mass Index; BIP, Body Image Perception; BIS, Body Image Satisfaction; ^∗^*p* < 0.01. The bold values indicate Fisher’s Z equal or greater to 2.33.*

**TABLE 7:** Pearson’s correlation and Fisher’s *Z* between variables of second-order interaction effects of the linear regression analysis shown in Table 5.

		**Anxious/depressed**	**Withdrawn/depressed**	**Somatic complaints**	**Internalizing symptoms**
**Correlation according to sex and age**		**Boys/Girls**	**Boys/Girls**	**Boys/Girls**	**Boys/Girls**

BMI	13–14 years		–	0.165^∗^/0.161^∗^	–
				*Z* = 0.06	
	15–16 years	–		−0.010/0.140^∗^	
				*Z* = **3.97**	
	17–18 years			0.056/0.107^∗^	
				*Z* = 1.06	
BIP	13–14 years	–	–	–	–
	15–16 years				
	17–18 years				
BIS	13–14 years	–	–	–	–
	15–16 years				
	17–18 years				

**Correlation according to sex and FAS**		**Boys/Girls**	**Boys/Girls**	**Boys/Girls**	**Boys/Girls**

BMI	Low FAS	–	–	–	–
	Medium FAS				
	High FAS				
BIP	Low FAS	0.194^∗^/0.100	0.160^∗^/0.107	–	0.139^∗^/0.113
		*Z* = 1.47	*Z* = 0.83		*Z* = 0.40
	Medium FAS	0.030/0.196^∗^	0.073/0.149^∗^		0.037/0.166^∗^
		*Z* = **4.06**	*Z* = 1.85		*Z* = **3.14**
	High FAS	−0.015/0.207^∗^	−0.017/0.199^∗^		−0.052/0.246^∗^
		*Z* = **4.38**	*Z* = **4.25**		*Z* = **5.90**
	Low FAS	−0.464^∗^/−0.440^∗^	−0.451^∗^/−0.350^∗^	−0.251^∗^/−0.282^∗^	−0.426^∗^/−0.399^∗^
		*Z* = 0.46	*Z* = 1.84	*Z* = 0.51	*Z* = 0.48
BIS	Medium FAS	−0.361^∗^/−0.475^∗^	−0.340^∗^/−0.419^∗^	−0.231^∗^/−0.331^∗^	−0.342^∗^/−0.431^∗^
		*Z* = **3.33**	*Z* = 2.22	*Z* = **2.62**	*Z* = **2.52**
	High FAS	−0.317^∗^/−0.460^∗^	−0.288^∗^/−0.411^∗^	−0.217^∗^/−0.370^∗^	−0.269^∗^/−0.484^∗^
		*Z* = **3.29**	*Z* = **2.73**	*Z* = **3.27**	*Z* = **4.91**

**Correlation according to FAS and age**		**FAS: Low/Medium/High**	**FAS: Low/Medium/High**	**FAS: Low/Medium/High**	**FAS: Low/Medium/High**

BMI	13–14 years	–	–	–	–
	15–16 years				
	17–18 years				
BIP	13–14 years	–	–	–	–
	15–16 years				
	17–18 years				
BIS	13–14 years	–	–	–	–
	15–16 years				
	17–18 years				

*FAS, Family Affluence Scale; BMI, Body Mass Index; BIP, Body Image Perception; BIS, Body Image Satisfaction; ^∗^*p* < 0.01. The bold values indicate Fisher’s Z equal or greater to 2.33.*

Closely analyzing the first-order interaction effects (see Table 6), BMI was found to predict somatic complaints only in girls (*r* = 0.138), but not in their male peers (*r* = 0.041). However, delving deeper and considering the second-order interaction effects, upon combining the influence of sex and age, BMI showed a double inequality with respect to its influence on somatic complaints. Specifically, the positive relationship between BMI and somatic complaints occurred both in boys and girls at 13–14 years old, without differences between them, with a Pearson’s correlation of 0.165 and 0.161, respectively. However, from 15 years old onward a relationship between BMI and somatic complaints was only found in the case of girls (see Table 7).

Regarding the predictive capacity of BMI on anxious/depressed, the first-order interaction effects showed this relationship in 13–14 year-old adolescents (*r* = 0.207) but not in the other age groups (see Table 6). The relationship between BMI and the global scale of IS was found to be stronger in 13–14 year-olds (*r* = 0.274) than at later ages, although in this case the relationship continued to be significant for 15–16 year-olds (*r* = 0.147) and for 17–18 years old (*r* = 0.164). Lastly, in the case of affluence level, it was found that BMI had a predictive capacity on anxious/depressed only in the case of the adolescents with a low FAS score (*r* = 0.164), but not in those with a medium or high score.

The results regarding the first- and second-order interaction effects of overweight BIP on the internalizing indicators will be described next. Table 6 shows that the positive relationship between overweight BIP and all IS indicators were higher in girls. In fact, regarding somatic complaints and in the total of IS, this relationship with overweight BIP only occurred in girls. However, analysis of second-order interaction effects revealed that sex differences in the relationship between BIP and internalizing indicators varied according to FAS score. Specifically, Table 7 shows no sex differences in the relationship between overweight BIP and internalizing indicators in low FAS. In fact, in this affluence level it is boys rather than girls who show a relationship between body perception and IS. By contrast, in medium and high affluence only the girls demonstrated a relationship between overweight BIP and internalizing indicators. This relationship was stronger in high FAS, where the correlation reached 0.207 for anxious/depressed, 0.199 for withdrawn/depressed, and 0.246 for the global scale.

Lastly, the interaction effects also provided relevant information regarding the predictive capacity of BIS on internalizing indicators. Table 6 shows that, although age and FAS do not influence the aforementioned relationship, the adolescents’ sex does affect the negative relationship between BIS and withdrawn/depressed showing to be stronger in girls (*r* = −0.411) than in boys (*r* = −0.350). In addition, when sex differences in the relationship between BIS and internalizing indicators were analyzed –keeping in mind differences in FAS– differences were found in all internalizing indicators revealing new, previously undetected relationships, not only in withdrawn/depressed. Specifically, Table 7 shows a relationship between higher BIS and fewer internalizing indicators both in boys and girls when the adolescents had lower FAS. However, sex differences emerged as the family affluence increased. Thus, in adolescents with a high affluence, although the negative relationship between BIS and internalizing indicators kept occurring both in boys and in girls, this relationship was significantly higher in girls than in boys. For example, whereas the relationship between body image and the global scale of IS was −0.269 in boys, this relationship increased to −0.484 in the case of girls.

### The Role of BMI, Overweight BIP, Dieting, Physical Activity, and Sociodemographic Variables on the IS of Adolescents With Low BIS

Regarding the second research objective, this third section of the results focused on those adolescents with lower scores in the more significant body image component for IS. Specifically, BIS was the most important component in this relationship for the following two reasons: (a) on the bivariate level, satisfaction was the body image component that related most with the internalizing symptoms, as shown in Table 3; and (b) satisfaction with body image had a higher predictive capacity on IS in the linear regression, as shown in Table 5.

Therefore, we will next elaborate on the analyses of those adolescents with lower BIS, who represent 36.7% of the sample. For this objective, the sample was first divided into terciles according to body satisfaction (as shown in the rows of Table 8) and these adolescents were subsequently divided into three groups according to their level in the global scale of IS (as shown in the columns of Table 8).

**TABLE 8:** Sample subgroups (N and percentage) according to their tercile position in the internalizing scale of YSR and the BIS.

		**Internalizing symptoms (total)**		
	
		**Low (0–12)**	**Medium (12.50–20)**	**High (20.50–68)**
BIS	Low (1–3.6)	401 (8.0%) -*Group 1*-	459 (9.1%)	983 (19.6%) -*Group 2*-
	Medium (3.7–4.5)	708 (14.1%)	687 (13.7%)	453 (9.0%)
	High (4.6–5)	685 (13.6%)	444 (8.8%)	203 (4.0%)

*BIS, Body Image Satisfaction. The values shaded in gray correspond to the groups selected for the next analysis.*

Through this process, by selecting the extreme groups we were able to identify two groups of adolescents who, while in both cases showing low BIS, were differentiated amongst themselves by the low or high effect on IS. Specifically, Group 1 has low BIS and a low IS level (representing 8% of the sample), whereas Group 2 has low BIS and high levels of IS (19.6% of the sample).

Table 9 shows the differences between Groups 1 and 2 in the other research variables: sociodemographic variables, BMI, overweight BIP, dieting behaviors, and physical activity. In general terms, differences were found between the two groups in sex, age, overweight BIP, and physical activity. Specifically, there was a higher propensity to fall into Group 2, that is to say, the group of adolescents unsatisfied with their body image and who also had IS: girls (71.2% of adolescents in group 2); older (31.8% were 17–18 years old); those that perceived themselves as overweight (67.71%); and those that did not do any physical exercise or did so with low frequency, both in the case of moderate-vigorous (88.3%) and vigorous (79.1%) physical activity.

**TABLE 9:** Percentages (N), significance test, effect sizes, and logistic regression models on sample subgroups with low BIS comparing low (group 1) and high (group 2) presence of IS.

		**Descriptive statistics**				**Bivariate significance tests**	**Logistic regression**
				
		**Group 1**		**Group 2**			
					
		**% (N)**	***SR*_*C*_**	**% (N)**	***SR*_*C*_**		***R^2^* = 15.7%**
Sex	Boys (ref.)	51.4%(206)	**8.1**	28.8%(283)	**−8.1**	*X*^2^(1) = 63.57, *p* < 0.001; *phi* = **0.214**	B-G: OR = **2.83** (2.09 – 3.84)
	Girls	48.6%(195)	**−8.1**	71.2%(700)	**8.1**		
Age (years)	13–14 year (ref.)	22.2%(89)	**4.3**	12.7%(125)	**−4.3**	*X*^2^(2) = 26.44, *p* < 0.001; *V* = **0.138**	13–15: OR = **1.71** (1.16 – 2.51) 13–17: OR = **2.58** (1.69 – 3.93)
	15–16 year	50.9%(204)	1.2	49.4%(486)	–1.2		
	17–18 year	26.9%(108)	**−4.4**	37.8%(372)	**4.4**		
FAS	Low (ref.)	23.2%(82)	–0.1	24.5%(218)	0.1	*X*^2^(2) = 0.66, *p* = 0.719; *V* = 0.023	L-M: OR = 1.11 (0.77 – 1.59) L-H: OR = 1.03 (0.69 – 1.53)
	Medium	47.5%(168)	–0.6	48.3%(430)	0.6		
	High	29.4%(104)	0.8	27.2%(242)	–0.8		
BMI	Thin	14.3%(49)	2.2	9.9%(87)	–2.2	*X*^2^(2) = 5.02, *p* = 0.081; *V* = 0.064	O-T: OR = **0.49** (0.28 – 0.89) O-N: OR = 0.85 (0.59 – 1.24)
	Normal	62.3%(213)	–0.6	64.4%(565)	0.6		
	Overweight/obese (ref.)	23.4%(80)	–0.9	25.7%(226)	0.9		
BIP	Thin	13.4%(53)	–0.8	14.8%(145)	0.8	*X*^2^(2) = 21.25, *p* < 0.001; *V* = **0.124**	O-T: OR = 1.36 (0.82 – 2.27) O-N: OR = **0.60** (0.41 – 0.86)
	Normal	29.2%(116)	**5.1**	18.0%(176)	**−5.1**		
	Overweight (ref.)	57.4%(228)	**−3.8**	67.1%(656)	**3.8**		
Diet to lose	No (ref.)	76.5%(306)	0.3	76.4%(749)	–0.3	*X*^2^(1) = 0.01, *p* = 0.977; *phi* = 0.001	N-Y: OR = 0.88 (0.62 – 1.26)
	Yes	23.5%(94)	–0.3	23.6%(231)	0.3		
Diet to gain	No (ref.)	96.3%(385)	–0.3	96.5%(946)	0.3	*X*^2^(1) = 0.07, *p* = 0.798; *phi* = 0.007	N-Y: OR = 1.06 (0.49 – 2.27)
	Yes	3.8%(15)	0.3	3.5%(34)	–0.3		
MVPA	Null (ref.)	6.5%(24)	**−4.0**	16.0%(146)	**4.0**	*X*^2^(2) = 57.71, *p* < 0.001; *V* = **0.221**	N-L: OR = **0.48** (0.26 – 0.86) N-H: OR = **0.28** (0.14 – 0.55)
	Low	66.4%(245)	–1.9	72.3%(661)	1.9		
	High	27.1%(100)	**6.0**	11.7%(107)	**−6.0**		
VPA	Null (ref.)	13.2%(49)	–2.3	19.5%(177)	2.3	*X*^2^(2) = 12.98, *p* = 0.002; *V* = **0.101**	N-L: OR = 0.93 (0.59 – 1.44) N-H: OR = 0.93 (0.55 – 1.58)
	Low	57.8%(214)	–1.3	59.6%(542)	1.3		
	High	28.9%(107)	**3.5**	21.0%(191)	**−3.5**		

*The bold values indicate small (0.10–0.29), medium (0.30–0.49), or high (0.50 and above) effect size values – phi and V-, odd ratios which value 1 is outside the confidence interval and corrected standardized residuals –SRc– higher to 2.33 (one-sided confidence interval of 99%).*

Lastly, the last column of Table 9 shows the odds ratio (OR) analysis obtained from the logistical regression model highlighting the difference in the probability that the adolescents would pertain to Group 2, according to the aforementioned variables. This analysis showed a high predictive capacity, specifically 15.7%. Selecting only those OR in which the value 1 falls out of the confidence interval, a risk and protection profile was obtained based on the analyzed variables. Thus, in order of importance, the identified risk factors for pertaining to Group 2 were: be a girl (OR = 2.83), be aged 17–18 compared to 13–14 (OR = 2.58), and be 15–16 compared to 13–14 (OR = 1.71). By contrast, protective factors included a high level of moderate-VFA compared to none (OR = 0.28), the low level of this physical activity compared to none (OR = 0.48), have a BMI corresponding to thinness compared to overweight/obese (OR = 0.49), and perceiving oneself as a normal size compared to perceiving oneself as overweight (OR = 0.60).

## Discussion

### Problems With Body Image and Adolescence

Adolescence is an important stage for developing problems related to physical appearance, which can seriously affect the health of this population (Shagar et al., 2017). This present study –based on more than 4500 adolescents– shows body image to be a key aspect of their mental health. Specifically, considering the research objective of determining the predictive capacity of sociodemographic and body image variables on IS, in the second section of the results it was demonstrated that the 23.5% variability of IS in this sample was explained by the physiological (BMI), cognitive (BIP), and emotional (BIS) components of body image, as well as through the influence of sex, age, and FAS. More specifically, the anxious/depressed component was the dimension better explained by the aforementioned variables (25.3%), from amongst the four specific internalizing indicators examined and the global scale of IS.

Regarding the second research objective, explained in the third section of the Results, analyses focused on those adolescents with lower scores in the more significant body image component for IS. These results demonstrate that beyond BMI, the cognitive-emotional factors of body image –BIP and BIS– had a higher predictive capacity on mental health during adolescence. In fact, BIS was the body image dimension most strongly related to IS.

The results related to the central role that BIS has in adolescent health are congruent with findings from other research (Stice et al., 2003; Neumark-Sztainer et al., 2006; Ramos et al., 2012). For example, Rotenberg et al. (2004) noticed the difficulty of modifying aspects related to the subjective state of mind, despite achieving changes in the cognitive area with regards to body image. In addition, these results are congruent with studies that identify the self-imposition of having a body that is attractive to others as the root of body image related health problems, which is indicative of the need of people to be socially accepted (Sobrino-Bazaga and Rabito-Alcoón, 2018).

Therefore, in applying these results to intervention, it would appear critical to address factors related to self-acceptance and self-esteem. In fact, despite the importance of adequate cognitive restructuring in those adolescents who tend to perceive themselves as more overweight than they really are, the emotional component showed to have the highest predictive capacity in explaining internalizing symptomology. Therefore, based on our results it would be advised to prioritize this emotional component in intervention and prevention of body image disorders.

### Problems With Body Image in Adolescence, the Moderation of Sex and the Double Inequality Sex-Age

In recent years, various systematic reviews have highlighted the selection bias that exists in much of the research in this field (Larsen et al., 2015; Sobrino-Bazaga and Rabito-Alcoón, 2018), criticizing the higher level of attention on the feminine sex in studies on problems related to body image. On account of this, the results of the systematic reviews should be interpreted with caution due to the over-representation of females in the samples analyzed. In the present study, conducted with an equal representation of both sexes (2265 boys and 2266 girls), clear sex differences were found in problems related to body image, IS, and the combination of the two. To begin with, and coinciding with other studies (Barker and Galambos, 2003; McCabe and Ricciardelli, 2004; Larson et al., 2009; Kanaan and Afifi, 2010; Ramos et al., 2012, 2013), the bivariate results presented in the first section of the Results showed that girls present a higher frequency than boys to see themselves as fat, be dissatisfied with their body image, and diet to lose weight/volume.

Sex inequalities were also found regarding psychopathological problems related to IS, as was also found in the first section of the Results dedicated to determining the bivariate relationships between the studied variables. Congruent with other studies (Hayward and Sanborn, 2002; Rutter et al., 2003; Chaplin and Aldao, 2013; Oliva et al., 2015), adolescent girls had higher scores in all the subscales and in the global scale of IS. Likewise, regarding the last objective of this study, focused on the adolescents with lower scores in the more significant body image component for IS, the results showed that sex was also the most important factor in the presence of IS amongst adolescents with low BIS.

It is precisely in this specific area –the differences between adolescent boys and girls in the relationship of body image problems to IS– where fewer studies exist. Therefore, this research offers interesting information upon finding that the variable sex moderates the relationship between body image and mental health. Specifically, it is in the cognitive component (BIP) – amongst all the aspects of body image– where more differences exist between boys and girls in the relationship with IS. Thus, findings showed a stronger relationship in girls between overweight BIP and having IS. In this sense, Harter (2006) explained that many girls worship an ideal of thinness, overestimate the preferences of boys for slender female bodies, see themselves as fatter than other girls and negatively compare themselves with the models of women on display in the media. In addition, the results of this present study are not surprising in light of other research that shows how girls are more vulnerable to certain negative thought patterns found in depression, such as self-blame, a negative social comparison, hyper-vigilance to potential stress and brooding (that is to say, an obsession about the future consequences of their hypothetical decisions) (Hyde et al., 2008; Andrews and Thompson, 2009).

Regarding age, the bivariate results presented in the first section of the Results demonstrated that younger adolescents –between 13 and 14 years old– showed higher BIS and a lower IS. However, based on data from the last section of the Results, focused on analyzing the profile of those adolescents with low BIS, this age group also show a higher probability of suffering IS when they have low BIS. The reason for younger adolescents’ higher vulnerability could be due to the increased accumulation or simultaneousness of different developmental transitions at this time, such as puberty, changes in peer groups, parental relationships, or an increase in school demands. According to Brooks-Gunn (1991) the amount of stressful events increases during the beginning and middle of adolescence, and later decreases. Most adolescents transition positively through this period, however for some the coping skills could be overwhelmed by multiple life changes very close together, which would increase IS. In fact, the most vulnerable group in our study (low BIS and IS) represents only 19.6% of the sample. However, it would be opportune for future research to contrast these results with longitudinal data, which would permit one to analyze if in fact body dissatisfaction is a predecessor to IS in this developmental moment. In this sense, differential sensitivity models (Graber and Brooks-Gunn, 1996) or diathesis-stress models (Ingram and Luxton, 2005) could be compared, which refer to how individuals with specific preexisting characteristics are potentially more sensitive to developing problems in periods of transition and change.

Moreover, this study analyzed double inequality effects combining sex and age based on the analysis of second-order interaction effects shown in the second section of the Results. These results showed that whereas BMI predicted psychosomatic complaints in both boys and girls at 13–14 years old, the association among adolescents older than 15 years old was significant only among girls. There could be a biological explanation behind these sex inequalities, since the relationship is only found in the physiological component of body image (BMI), and not in the cognitive (BIP) or emotional components (BIS). In fact, among adolescents 15 years old and up, girls are more likely to receive medical attention in comparison with boys (Ozer and Irwin, 2009), something assistance services should keep in mind in. In this regard, AbouZahr (2014, p. 3) states: “paying due attention to the health of girls and women today is an investment not just for the present but also for the future and for future generations.”

### Problems With Body Image in Adolescence, the Moderation of FAS, and the Double Inequality Sex-Socioeconomic Level

Despite the importance of socioeconomic factors in the health of children and adults having been widely demonstrated (e.g., Marmot and Bell, 2012), there is little evidence on the impact of socioeconomic inequalities on adolescent health (Viner et al., 2012). However, inequalities in overweightness and obesity during adolescence have been stated (O’Dea and Caputi, 2001; Cheung et al., 2011; Pereira et al., 2011). In close relationship to the findings of these prior studies, the bivariate results of this present research shown in the first section of the Results, reveal a higher BMI in adolescents with low FAS compared to their peers with high FAS.

In explanation of these results, significant associations have been found between FAS and eating breakfast, fruit, vegetables, and candy (Fismen et al., 2012; Vereecken et al., 2015; Lazzeri et al., 2016; Yannakoulia et al., 2016; Moreno-Maldonado et al., 2018). Food access seems to be central in the relationship between family socioeconomic level and the type of food consumed. Turrell et al. (2003) points out that economically disadvantaged parents are less likely to buy healthy foods. Ward et al. (2013) found that families with lower incomes would have to spend approximately 30% of their family income to heating healthily, whereas families with higher income would spend around 10%.

Results from previous research examining socioeconomic inequalities in the cognitive-emotional component of body image are less consistent. For example, whereas a study conducted by Cok (1990) found no association between BIS and SES, Pereira et al. (2009) found a relationship between lower BIS due to being overweight and adolescents with low SES, and in a study conducted by Wang et al. (2005) results showed an opposite relationship: adolescents with a higher SES showed lower BIS. The incongruences found in the previous studies might be due to differences in the place of origin of the samples used. Studies conducted in developed countries tend to show that adolescents with a higher SES and better access to social media –where thinness is shown as a beauty ideal– are less satisfied with their body image than their peers from a lower SES (Wang et al., 2005; Pereira et al., 2011; Feng and Wilson, 2016). However, the results of our current study showed, in the first section of the Results focused on the bivariate analyses, that adolescents with a low FAS score more frequently demonstrated an overweight BIP and have low BIS. This discrepancy could be explained by the high correlation found between the variables BMI, overweight BIP, and low BIS. However, other interesting explanations could be found in the double inequality effect that happens between socioeconomic level and the adolescents’ sex in how their body image affects their mental health.

In this regard, our research showed in the second section of the Results, and more specifically in the second-order interaction effects, that the relationship between overweight BIP and IS was only significant among boys with low FAS. In contrast, the association was found in all socioeconomic levels in the case of girls, being even stronger for girls pertaining to families with a medium/higher SES. Likewise, the association between low BIS and IS did not show differences among boys and girls pertaining to families with low FAS. However, sex differences were found in high FAS, with adolescent girls demonstrating a stronger association than boys between low BIS and IS.

Although little research has taken into account the double inequality perspective, a previous study conducted by Story et al. (1995) showed that girls with lower socioeconomic levels have a lower probability of perceiving themselves as fat. Likewise, O’Dea and Caputi (2001), based on research with 1131 Australian children and adolescents between 6 and 19 years old, pointed out that the youth with lower socioeconomic level, especially males, could be particularly resistant to sociocultural influences of thinness as an ideal of beauty. However, considering the presence of indigenous people in Australia one should be cautious when generalizing these results. It would be opportune for future to control aspects related to the ethnicity of the studied population.

### The Role of Weight and Body Image Behaviors in the Presence of Internalizing Symptoms

As was discussed in the Introduction section, and in consonance with the RDS metatheory, this research focused not only on the biological, psychological, and emotional components of body image, but also on its behavioral dimensions in considering dieting behavior and physical activity.

Regarding dieting behaviors, the first section of the Results showed the bivariate relationship of this type of variable and the rest of the studied variables. This research found that boys, older adolescents, and those pertaining to families with higher FAS more frequently diet to gain weight/volume. However, sex inequalities were less pronounced with respect to dieting to lose weight/volume nor were there age or socioeconomic differences. Therefore, an interesting finding from this research, within these bivariate analyses, was that whereas boys were clearly more likely than girls to diet to gain weight, the differences between them were less pronounced in dieting to lose weight. These results are congruent with a recent study (Chu et al., 2019) that showed a greater pressure in general on boys to lose weight as well as to gain weight. Although it seems paradoxical, it appears that boys feel pressure to gain weight and muscles, as well as pressure to lose weight for leanness.

Attending to the higher frequency of dieting to gain weight/volume as age increases, these results are found in the first section of the Results dedicated to bivariate analysis. These results are congruent with other studies that report an increased pressure to be muscular throughout this developmental stage, which is especially noticeable in the case of boys. For example, in a study conducted by McCabe and Ricciardelli (2004) with 800 Australian adolescents, boys were almost equally divided between wanting to lose weight and wanting to gain muscle size. In addition, a prospective study conducted over the course of 1 year by Shomaker and Furman (2010) demonstrated that adolescent boys had higher scores on driving for muscularity and preoccupation with muscularity. This pressure might be stronger for adolescents from a higher SES, which would explain our bivariate results indicating that adolescents with high FAS were more likely to diet to gain weight/volume than adolescents with low FAS. For example, a comprehensive research previously exploring differences in dieting behaviors in almost 10000 adolescents from different socioeconomic backgrounds conducted by Neumark-Sztainer et al. (1999) found that, besides weight preoccupation being prevalent among all groups, adolescents from more affluent families dieted and exercised more to gain weight, which supports our findings. However, the same study also showed that adolescents pertaining to less affluent families presented more disordered eating and behaviors such as taking food supplements to gain weight or muscle.

Adolescence marks a time of increased independence about food choices and consumption. Among those adolescents who are dissatisfied with their body image, some will undertake weight control methods. In fact, in the last section of the Results, the relationship between low BIS and dieting to lose weight/volume –which was significant for both adolescents with IS (23.6% of the adolescents) as well as those without these symptoms (23.5% of the adolescents)– but not with dieting to gain weight/volume. These findings are important, given that medical monitoring is generally absent during dieting (Eisenberg et al., 2005; Larson et al., 2009) and the weight-reduction behaviors that adolescents undertake are not always appropriate (Timlin et al., 2008; Ramos et al., 2013) and often results in a weight gain and poorer overall health (Neumark-Sztainer et al., 2006).

With respect to physical activity, its relationship with weight has been extensively studied, and the global reduction in physical activity has been identified as a possible cause of the increase in obesity among western societies (Janssen et al., 2005). Short-term intervention trials also indicate that physical activity is the most effective weight control strategy (McGuire et al., 1999).

The bivariate results outlined in the first section of the Results showed that girls, older adolescents, and those with low FAS scores present less psychical activity in both measures examined (MVPA and VPA). It is commonly known that boys associate sports with masculinity and believe they earn prestige through competition, whereas girls are less likely to relate a sports activity with femininity and may avoid participating in those activities that could threaten their femininity (Coakley and White, 1992). Likewise, as Inchley and Currie (2004) explain, historically males have always had fewer restrictions on their activities compared to females, for example in many contexts boys get permission to be out of the house unsupervised with more frequency than girls.

In addition, just like in the aforementioned research by Neumark-Sztainer et al. (1999), other studies (Borraccino et al., 2009; Stalsberg and Pedersen, 2010) have demonstrated socioeconomic inequalities in doing physical activity. Thus, it is important to emphasize the importance of implementing and carrying out public policies that make sports more affordable, as well as facilitate resources and infrastructure, so that they can become a daily practice in all layers of society.

In the first part of this study, dedicated to the bivariate results, an association was also found between a higher frequency of physical activity and higher BIS, and with a lower presence of IS. Along these lines Hausenblas and Fallon (2006) and Holmqvist and Frisen (2019) found that regular physical activity is a protective factor against the development of body image problems. In fact, the results of our study found that even a low frequency of physical activity is beneficial in comparison to its complete absence. Likewise, in the logistical regression shown in the last part of the Results, it was found that MVPA, even on a low level, is a protective factor amongst adolescents with low BIS, who more frequently show IS.

These results are especially relevant for intervention given the health promotion recommendations to increase healthy food consumption and the frequency of physical activity for long-term body weight control and to maintain a healthy weight (Centers for Disease Control and Prevention, 1997; European Commission for Health, and Consumer Protection, 2003). However, according to the *displacement hypothesis* (Andersen et al., 1998), it is increasingly difficult for adolescents to dedicate time to physical activity given the increase in sedentary activities in this developmental stage, such as in time dedicated to school work or recreation related to small screens like watching videos or playing videogames. Therefore, these sedentary activities would compete for the adolescents’ available time for physical activity. However, the fact that our study has demonstrated that a low frequency of physical activity (compared to none) had a higher predictive capacity on internalizing symptoms of the adolescents with low BIS, gives hope that this behavior could really be carried out during this developmental stage on a beneficial level. In this sense, health programs trying to promote physical activity in adolescents –as part of a healthy lifestyle– should take into consideration this question related to the effectiveness of physical activity also on a low level.

### Limitations and Strengths of This Study

This study has some limitations that should be taken into consideration when interpreting its results. Firstly, the cross-sectional design means that the results must be interpreted on an associative level, and it is not possible to draw conclusions about the directionality of the relationships found. Secondly, some methodological limitations should be mentioned. Several variables were assessed by frequency, not quantity. For example, VPA was measured with a seven-point Likert scales but recoded into a three-level categorical variable to facilitated data analysis. This reduced information diversity. Thirdly, it would be appropriate to include different contextual factors in the analysis. Further research should explore how family, peers, and school environment influence adolescent body image and the relationship with internalizing symptoms. Fourthly, self-reported BMI was used as an anthropometric measure, which may lead one to question its validity. However, previous studies have shown an elevated coincidence between BMI information provided by adolescents and the real measures taken on their weight and size. For example, Strauss (1999) reported more than a 94% coincidence between the information provided by 12–16 year old youth regarding their BMI and the scores obtained through precise and objective measures. Nonetheless, all the variables in this study were self-reported, which could imply a mono-method bias. Therefore, it could be convenient for future research to replicate these analysis based on measures obtained from other informants, not only in aspects such as BMI, but also in other questions such as, for example, the SES of the family.

Despite the aforementioned limitations, this study also has important strengths. This research offers information about a large and representative sample. Specifically, the sample selection was conducted under strict methodological guidelines, obtaining a representative sample of school-aged adolescents by age, type of school (public or private), and region of Spain. This size and representativeness support our recommendations for practical implications derived from the thoroughly discussed results.

In addition, the instruments used add to the strength of this research. On one hand, as has been explained in the Method, evaluation measures from classic and internationally acclaimed adolescent research, like the HBSC, have been used. On the other, this study is innovative in its addition of the YSR, a very exhaustive instrument currently recognized in psychopathological evaluation. This evaluation instrument uses a dimensional approach when assuming that the adolescents’ behaviors, as part of a continuum, could vary in their degree as well as be condensed in groups of symptoms or problems (Achenbach and Rescorla, 2001). Thus, by using this instrument this study has avoided adopting a purely taxonomic approach of mental health disorders, which has shown a higher identification of false positives (Helzer et al., 2008).

Finally, this research not only considered representative variables of all body image dimensions and used a validated instrument to evaluate internalizing symptoms, but in addition their associations were analyzed to observe the effects of sex, age, and socioeconomic inequalities on their relationship allowing double social inequalities to be detected.

## Conclusion

Based on a sample and quality evaluation instruments, this paper has identified the roles of sex, age, and FAS in the relationship between body image problems and IS in a representative sample of adolescents. On one hand, of the three dimensions in the scale of IS (anxious/depressed, withdrawn/depressed, and somatic complaints), it is anxious/depressed that is best explained by the combination of BMI, overweight BIP, BIS, sex, age, and FAS. On the other hand, between the different components of body image, BIS showed a higher predictive capacity both on the three subscales and on the global scale of IS.

In addition, the large sample size used in this study allowed our analyses to detect possible double inequality effects. For example, this study found that whereas boys are more vulnerable in the high relationship between body image problems and IS when they have low FAS, in the case of girls they are most vulnerable in high FAS. These results require prevention programs and interventions to adapt to the specific combination of sociodemographic characteristics of the people with whom they work.

On the other hand, this study has found interesting results regarding the role that body image behaviors have, such as dieting to lose or gain weight/volume, and physical activity, when analyzing in detail those adolescents with worse scores in BIS. In this sense, a strong relationship is found between low BIS and dieting to lose weight/volume that occurs independently from whether the adolescents show IS or not. Keeping in mind the low nutritional quality of diets that adolescents usually do (as has been discussed above), it is indispensable to prevent them from adopting these weight control behaviors as well as promote eating habits that achieve weight control in a healthier way. Likewise, this study finds that even a low frequency of physical activity, compared to none, is beneficial and protects adolescents against IS. This finding also supports interventions focused on this area, which may be able to set less demanding and more achievable goal since adolescents generally have to juggle many activities simultaneously.

## Data Availability

The datasets for this study will not be made publicly available because the data belong to the HBSC study network. The Spanish Ministry of Health, Social Services and Equality has a link to request the dataset in http://www.mscbs.gob.es/, within the following links: Sanidad – Profesionales – Salud pública – Prevención y Promoción – Promoción de la salud – Salud de los Jóvenes. Requests to access the datasets should be directed to CM: mcmoreno@us.es.

## Ethics Statement

The protocol was approved by the “Comité de Ética de la Investigación en Andalucía (Sistema Sanitario Público Andaluz).” Informed consent was obtained in accordance with the Declaration of Helsinki.

## Author Contributions

All authors conceived of the study, participated in its design and helped to draft the manuscript. Likewise, all authors made suggestions and critical reviews to the initial draft and contributed to its improvement until reaching the final manuscript, which was read and approved by all authors.

## Conflict of Interest Statement

The authors declare that the research was conducted in the absence of any commercial or financial relationships that could be construed as a potential conflict of interest.
